# *N*-Phenyl­anthranilic anhydride

**DOI:** 10.1107/S1600536809013002

**Published:** 2009-04-18

**Authors:** Guan-Feng Liu, Yong-Wen Luo, Da-Bin Qin

**Affiliations:** a School of Chemistry and Chemical Engineering, China West Normal University, Nanchong 637002, People’s Republic of China

## Abstract

The complete mol­ecule of the title compound, C_26_H_20_N_2_O_3_, is generated by crystallographic twofold symmetry, with the central O atom lying on the rotation axis. The conformation is stabilized by an intra­molecular N—H⋯O hydrogen bond. The dihedral angle between the inner and outer aromatic ring planes is 61.12 (5)°.

## Related literature

For the synthesis, see: Martín *et al.* (2006 ▶); Wiklund *et al.* (2004 ▶). For related structures, see: Duesler *et al.* (1981 ▶); Huelgas *et al. *(2006 ▶).
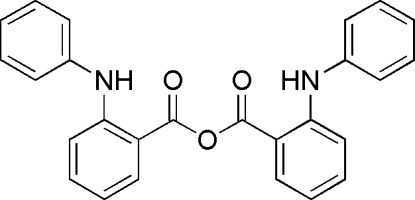


## Experimental

### 

#### Crystal data

C_26_H_20_N_2_O_3_
*M* *_r_* = 408.44
Monoclinic, 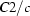

*a* = 9.090 (3) Å
*b* = 21.056 (6) Å
*c* = 10.623 (3) Å
β = 100.594 (3)°
*V* = 1998.5 (10) Å^3^
*Z* = 4
Mo *K*α radiation
μ = 0.09 mm^−1^
*T* = 93 K
0.33 × 0.30 × 0.18 mm

#### Data collection

Rigaku Spider diffractometer
Absorption correction: none
8102 measured reflections
2275 independent reflections
2000 reflections with *I* > 2σ(*I*)
*R*_int_ = 0.023

#### Refinement

*R*[*F* ^2^ > 2σ(*F* ^2^)] = 0.039
*wR*(*F* ^2^) = 0.111
*S* = 1.00
2275 reflections
145 parameters
H atoms treated by a mixture of independent and constrained refinement
Δρ_max_ = 0.25 e Å^−3^
Δρ_min_ = −0.20 e Å^−3^

### 

Data collection: *RAPID-AUTO* (Rigaku/MSC, 2004 ▶); cell refinement: *RAPID-AUTO*; data reduction: *RAPID-AUTO*; program(s) used to solve structure: *SHELXS97* (Sheldrick, 2008 ▶); program(s) used to refine structure: *SHELXL97* (Sheldrick, 2008 ▶); molecular graphics: *SHELXTL* (Sheldrick, 2008 ▶); software used to prepare material for publication: *SHELXTL*.

## Supplementary Material

Crystal structure: contains datablocks global, I. DOI: 10.1107/S1600536809013002/hb2927sup1.cif

Structure factors: contains datablocks I. DOI: 10.1107/S1600536809013002/hb2927Isup2.hkl

Additional supplementary materials:  crystallographic information; 3D view; checkCIF report

## Figures and Tables

**Table 1:** Hydrogen-bond geometry (Å, °)

*D*—H⋯*A*	*D*—H	H⋯*A*	*D*⋯*A*	*D*—H⋯*A*
N1—H1*N*⋯O1	0.903 (16)	1.966 (15)	2.6629 (14)	132.7 (13)

**Fig. 1.**
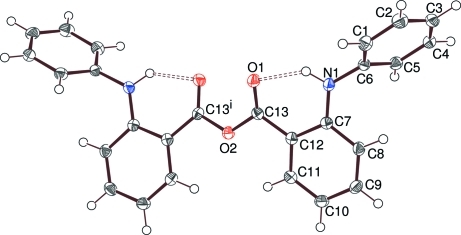
The molecular structure of (I) showing 50% probability displacement ellipsoids for the non-hydrogen atoms. Symmetry code: (i) –x, y, 1/2–y.

## supplementary crystallographic information

### Comment

*N*-Phenylanthranilic acid derivatives display a antipyretic acitivity, its RhI complex is known as a remarkably active hydrogenation catalyst. *N*-phenyl anthranilic acid anhydride, which is considered as an important reaction intermediate, We here report the crystal structure of the title compound, (I).

Bond lengths and angles in (I) (Fig. 1) are within their normal ranges. In each independent molecule, the arrangement of N—H···O hydrogen bond and the planar (O=C—O) group is almost coplanar with respect to its carrier benzene ring, with dihedral angle of 6.63 (1)°, but the two benzene rings in the diphenylamine units are twisted with a dihedral angle of 61.12 (4)°. The structure is stabilized by an intramolecular hydrogen bond from an H atom of a amido N atom to a carbonyl O atom on the six-membered ring.

### Experimental

The title compound was prepared according to the reported procedure of Martín *et al.* (2006) and Wiklund *et al.* (2004). Colourless chunks of (I) were obtained by recrystallization from ethyl acetate.

### Refinement

The N-bound N atom was located in a difference map and its position and U_iso_ value were freely refined. The C-bound H atoms were placed in calculated positions with C—H = 0.95 Å and refined as riding with *U*_iso_(H) = 1.2*U*_eq_(C).

### Crystal data

**Table:**

C_26_H_20_N_2_O_3_	*F*(000) = 856
*M**_r_* = 408.44	*D*_x_ = 1.358 Mg m^−^^3^
Monoclinic, *C*2/*c*	Mo *K*α radiation, λ = 0.71073 Å
Hall symbol: -C 2yc	Cell parameters from 3250 reflections
*a* = 9.090 (3) Å	θ = 3.4–27.5°
*b* = 21.056 (6) Å	µ = 0.09 mm^−^^1^
*c* = 10.623 (3) Å	*T* = 93 K
β = 100.594 (3)°	Chunk, colourless
*V* = 1998.5 (10) Å^3^	0.33 × 0.30 × 0.18 mm
*Z* = 4	

### Data collection

**Table:**

Rigaku Spider diffractometer	2000 reflections with *I* > 2σ(*I*)
Radiation source: Rotating Anode	*R*_int_ = 0.023
graphite	θ_max_ = 27.5°, θ_min_ = 3.4°
ω scans	*h* = −11→11
8102 measured reflections	*k* = −27→27
2275 independent reflections	*l* = −10→13

### Refinement

**Table:**

Refinement on *F*^2^	Primary atom site location: structure-invariant direct methods
Least-squares matrix: full	Secondary atom site location: difference Fourier map
*R*[*F*^2^ > 2σ(*F*^2^)] = 0.039	Hydrogen site location: inferred from neighbouring sites
*wR*(*F*^2^) = 0.111	H atoms treated by a mixture of independent and constrained refinement
*S* = 1.00	*w* = 1/[σ^2^(*F*_o_^2^) + (0.0706*P*)^2^ + 0.28*P*] where *P* = (*F*_o_^2^ + 2*F*_c_^2^)/3
2275 reflections	(Δ/σ)_max_ < 0.001
145 parameters	Δρ_max_ = 0.25 e Å^−^^3^
0 restraints	Δρ_min_ = −0.20 e Å^−^^3^

### Special details

**Table:**

Geometry. All e.s.d.'s (except the e.s.d. in the dihedral angle between two l.s. planes) are estimated using the full covariance matrix. The cell e.s.d.'s are taken into account individually in the estimation of e.s.d.'s in distances, angles and torsion angles; correlations between e.s.d.'s in cell parameters are only used when they are defined by crystal symmetry. An approximate (isotropic) treatment of cell e.s.d.'s is used for estimating e.s.d.'s involving l.s. planes.
Refinement. Refinement of *F*^2^ against ALL reflections. The weighted *R*-factor *wR* and goodness of fit *S* are based on *F*^2^, conventional *R*-factors *R* are based on *F*, with *F* set to zero for negative *F*^2^. The threshold expression of *F*^2^ > σ(*F*^2^) is used only for calculating *R*-factors(gt) *etc*. and is not relevant to the choice of reflections for refinement. *R*-factors based on *F*^2^ are statistically about twice as large as those based on *F*, and *R*- factors based on ALL data will be even larger.

### Fractional atomic coordinates and isotropic or equivalent isotropic displacement parameters (Å^2^)

**Table:**

	*x*	*y*	*z*	*U*_iso_*/*U*_eq_	
O1	0.11910 (9)	0.38137 (4)	0.34545 (8)	0.0229 (2)	
O2	0.0000	0.29421 (5)	0.2500	0.0225 (3)	
N1	0.41159 (11)	0.37188 (5)	0.43582 (9)	0.0204 (2)	
C1	0.56426 (13)	0.43835 (6)	0.59357 (11)	0.0236 (3)	
H1	0.4845	0.4399	0.6401	0.028*	
C2	0.69505 (14)	0.47174 (6)	0.63703 (12)	0.0275 (3)	
H2	0.7045	0.4964	0.7130	0.033*	
C3	0.81191 (14)	0.46930 (6)	0.57006 (13)	0.0274 (3)	
H3	0.9020	0.4918	0.6006	0.033*	
C4	0.79712 (13)	0.43398 (5)	0.45834 (12)	0.0246 (3)	
H4	0.8771	0.4324	0.4121	0.030*	
C5	0.66575 (13)	0.40089 (5)	0.41392 (11)	0.0215 (3)	
H5	0.6555	0.3771	0.3369	0.026*	
C6	0.54946 (12)	0.40262 (5)	0.48214 (11)	0.0185 (3)	
C7	0.39399 (12)	0.30870 (5)	0.40566 (10)	0.0176 (2)	
C8	0.51448 (12)	0.26579 (6)	0.43627 (11)	0.0207 (3)	
H8	0.6099	0.2811	0.4764	0.025*	
C9	0.49596 (13)	0.20234 (6)	0.40898 (11)	0.0233 (3)	
H9	0.5787	0.1744	0.4313	0.028*	
C10	0.35825 (13)	0.17796 (6)	0.34912 (11)	0.0234 (3)	
H10	0.3469	0.1339	0.3305	0.028*	
C11	0.23933 (13)	0.21874 (5)	0.31759 (11)	0.0206 (3)	
H11	0.1453	0.2025	0.2763	0.025*	
C12	0.25345 (12)	0.28396 (5)	0.34495 (10)	0.0179 (3)	
C13	0.12427 (12)	0.32625 (5)	0.31547 (10)	0.0183 (2)	
H1N	0.3269 (17)	0.3947 (7)	0.4330 (15)	0.039 (4)*	

### Atomic displacement parameters (Å^2^)

**Table:**

	*U*^11^	*U*^22^	*U*^33^	*U*^12^	*U*^13^	*U*^23^
O1	0.0191 (4)	0.0209 (4)	0.0273 (5)	0.0013 (3)	0.0006 (3)	−0.0033 (3)
O2	0.0175 (6)	0.0198 (6)	0.0272 (6)	0.000	−0.0037 (5)	0.000
N1	0.0157 (5)	0.0197 (5)	0.0248 (5)	0.0012 (4)	0.0014 (4)	−0.0022 (4)
C1	0.0231 (6)	0.0258 (6)	0.0215 (6)	0.0022 (5)	0.0033 (5)	−0.0004 (5)
C2	0.0299 (7)	0.0248 (6)	0.0244 (6)	0.0003 (5)	−0.0037 (5)	−0.0046 (5)
C3	0.0219 (6)	0.0198 (6)	0.0367 (7)	−0.0020 (4)	−0.0045 (5)	0.0005 (5)
C4	0.0199 (6)	0.0200 (6)	0.0340 (7)	0.0010 (4)	0.0051 (5)	0.0036 (5)
C5	0.0225 (6)	0.0199 (6)	0.0215 (6)	0.0012 (4)	0.0027 (5)	−0.0015 (4)
C6	0.0171 (6)	0.0170 (5)	0.0197 (5)	0.0008 (4)	−0.0009 (4)	0.0014 (4)
C7	0.0181 (6)	0.0189 (5)	0.0158 (5)	−0.0003 (4)	0.0033 (4)	0.0015 (4)
C8	0.0170 (6)	0.0234 (6)	0.0212 (6)	0.0002 (4)	0.0019 (4)	0.0035 (4)
C9	0.0218 (6)	0.0230 (6)	0.0256 (6)	0.0058 (4)	0.0059 (5)	0.0055 (5)
C10	0.0260 (6)	0.0179 (5)	0.0268 (6)	0.0004 (5)	0.0064 (5)	0.0007 (5)
C11	0.0206 (6)	0.0210 (6)	0.0203 (6)	−0.0019 (4)	0.0039 (4)	0.0002 (4)
C12	0.0181 (6)	0.0202 (6)	0.0157 (5)	0.0005 (4)	0.0037 (4)	0.0014 (4)
C13	0.0173 (6)	0.0197 (5)	0.0175 (5)	−0.0021 (4)	0.0023 (4)	0.0002 (4)

### Geometric parameters (Å, °)

**Table:**

O1—C13	1.2067 (14)	C4—H4	0.9500
O2—C13^i^	1.3877 (12)	C5—C6	1.3876 (17)
O2—C13	1.3877 (12)	C5—H5	0.9500
N1—C7	1.3708 (15)	C7—C8	1.4109 (15)
N1—C6	1.4159 (14)	C7—C12	1.4199 (15)
N1—H1N	0.903 (16)	C8—C9	1.3708 (17)
C1—C2	1.3853 (18)	C8—H8	0.9500
C1—C6	1.3880 (16)	C9—C10	1.3936 (17)
C1—H1	0.9500	C9—H9	0.9500
C2—C3	1.3838 (19)	C10—C11	1.3728 (16)
C2—H2	0.9500	C10—H10	0.9500
C3—C4	1.3860 (18)	C11—C12	1.4047 (15)
C3—H3	0.9500	C11—H11	0.9500
C4—C5	1.3881 (16)	C12—C13	1.4610 (15)
			
C13^i^—O2—C13	121.84 (12)	N1—C7—C8	121.02 (10)
C7—N1—C6	125.64 (9)	N1—C7—C12	121.25 (10)
C7—N1—H1N	116.5 (10)	C8—C7—C12	117.72 (10)
C6—N1—H1N	117.6 (10)	C9—C8—C7	121.02 (10)
C2—C1—C6	120.13 (11)	C9—C8—H8	119.5
C2—C1—H1	119.9	C7—C8—H8	119.5
C6—C1—H1	119.9	C8—C9—C10	121.37 (11)
C3—C2—C1	120.18 (11)	C8—C9—H9	119.3
C3—C2—H2	119.9	C10—C9—H9	119.3
C1—C2—H2	119.9	C11—C10—C9	118.80 (11)
C2—C3—C4	119.82 (11)	C11—C10—H10	120.6
C2—C3—H3	120.1	C9—C10—H10	120.6
C4—C3—H3	120.1	C10—C11—C12	121.54 (11)
C3—C4—C5	120.16 (12)	C10—C11—H11	119.2
C3—C4—H4	119.9	C12—C11—H11	119.2
C5—C4—H4	119.9	C11—C12—C7	119.53 (10)
C6—C5—C4	119.98 (11)	C11—C12—C13	120.82 (10)
C6—C5—H5	120.0	C7—C12—C13	119.62 (10)
C4—C5—H5	120.0	O1—C13—O2	122.15 (10)
C5—C6—C1	119.73 (10)	O1—C13—C12	126.76 (10)
C5—C6—N1	121.13 (10)	O2—C13—C12	111.05 (10)
C1—C6—N1	119.01 (10)		
			
C6—C1—C2—C3	−0.43 (18)	C8—C9—C10—C11	−0.18 (18)
C1—C2—C3—C4	0.82 (18)	C9—C10—C11—C12	−0.38 (17)
C2—C3—C4—C5	−0.28 (18)	C10—C11—C12—C7	0.54 (17)
C3—C4—C5—C6	−0.66 (17)	C10—C11—C12—C13	−177.48 (11)
C4—C5—C6—C1	1.05 (16)	N1—C7—C12—C11	−178.92 (10)
C4—C5—C6—N1	176.88 (10)	C8—C7—C12—C11	−0.14 (16)
C2—C1—C6—C5	−0.51 (17)	N1—C7—C12—C13	−0.87 (16)
C2—C1—C6—N1	−176.42 (10)	C8—C7—C12—C13	177.90 (10)
C7—N1—C6—C5	56.75 (16)	C13^i^—O2—C13—O1	18.14 (8)
C7—N1—C6—C1	−127.39 (12)	C13^i^—O2—C13—C12	−164.03 (10)
C6—N1—C7—C8	9.20 (17)	C11—C12—C13—O1	172.29 (11)
C6—N1—C7—C12	−172.07 (10)	C7—C12—C13—O1	−5.73 (18)
N1—C7—C8—C9	178.37 (10)	C11—C12—C13—O2	−5.43 (14)
C12—C7—C8—C9	−0.41 (17)	C7—C12—C13—O2	176.56 (9)
C7—C8—C9—C10	0.58 (18)		

Symmetry codes: (i) −*x*, *y*, −*z*+1/2.

### Hydrogen-bond geometry (Å, °)

**Table:**

*D*—H···*A*	*D*—H	H···*A*	*D*···*A*	*D*—H···*A*
N1—H1N···O1	0.903 (16)	1.966 (15)	2.6629 (14)	132.7 (13)

## References

[bb1] Duesler, E. N., Kress, R. B., Lin, C. T., Shiau, W. I., Paul, I. C. & Curtin, D. Y. (1981). *J. Am. Chem. Soc.* **103**, 875–879.

[bb2] Huelgas, G., Quintero, L., Anaya de Parrodi, C. & Bernès, S. (2006). *Acta Cryst.* E**62**, o3191–o3192.

[bb3] Martín, A., Mesa, M., Docampo, M. L., Gómez, V. & Pellón, R. F. (2006). *Synth. Commun.* **36**, 271–277.

[bb4] Rigaku/MSC (2004). *RAPID-AUTO* Rigaku/MSC Inc., The Woodlands, Texas, USA.

[bb5] Sheldrick, G. M. (2008). *Acta Cryst.* A**64**, 112–122. 10.1107/S0108767307043930 18156677

[bb6] Wiklund, P., Evans, M. R. & Bergman, J. (2004). *J. Org. Chem.* **69**, 6371–6376. 10.1021/jo049169b 15357597

